# Asthma Due to Maltese Cats

**Published:** 1913-11

**Authors:** 


Asthma Due to Maltese Cats. H. C. Wood, Jr., of Philadelphia, *Monthly Cyc. and Med. Bull.*, June, 1913, cites a case. The patient, a girl of 19, was subject to asthmatic attacks from other cause, but always had an attack precipitated by the presence of maltese, though not of other cats.

